# Subsurface Cooling Rates and Microstructural Response during Laser Based Metal Additive Manufacturing

**DOI:** 10.1038/s41598-020-58598-z

**Published:** 2020-02-06

**Authors:** Vivek Thampy, Anthony Y. Fong, Nicholas P. Calta, Jenny Wang, Aiden A. Martin, Philip J. Depond, Andrew M. Kiss, Gabe Guss, Qingfeng Xing, Ryan T. Ott, Anthony van Buuren, Michael F. Toney, Johanna Nelson Weker, Matthew J. Kramer, Manyalibo J. Matthews, Christopher J. Tassone, Kevin H. Stone

**Affiliations:** 1 Stanford Synchrotron Radiation Lightsource, SLAC National Accelerator Laboratory, Menlo Park, CA 94025 United States; 2 Physical and Life Sciences Directorate, Lawrence Livermore National Laboratory, Livermore, CA 94550 USA; 3 Engineering Directorate, Lawrence Livermore National Laboratory, Livermore, CA 94550 USA; 4 Division of Materials Science and Engineering, Ames Laboratory, Ames, IA 50011 USA

**Keywords:** Metals and alloys, Characterization and analytical techniques

## Abstract

Laser powder bed fusion (LPBF) is a method of additive manufacturing characterized by the rapid scanning of a high powered laser over a thin bed of metallic powder to create a single layer, which may then be built upon to form larger structures. Much of the melting, resolidification, and subsequent cooling take place at much higher rates and with much higher thermal gradients than in traditional metallurgical processes, with much of this occurring below the surface. We have used *in situ* high speed X-ray diffraction to extract subsurface cooling rates following resolidification from the melt and above the *β*-transus in titanium alloy Ti-6Al-4V. We observe an inverse relationship with laser power and bulk cooling rates. The measured cooling rates are seen to correlate to the level of residual strain borne by the minority *β*-Ti phase with increased strain at slower cooling rates. The *α*-Ti phase shows a lattice contraction which is invariant with cooling rate. We also observe a broadening of the diffraction peaks which is greater for the *β*-Ti phase at slower cooling rates and a change in the relative phase fraction following LPBF. These results provide a direct measure of the subsurface thermal history and demonstrate its importance to the ultimate quality of additively manufactured materials.

## Introduction

In LPBF, a common metal additive manufacturing (AM) process, a thin layer of precursor powder is selectively melted by tracing a high power laser to create a single, solid layer, followed by the spread of the next layer of powder for melting, and so on to build a part layer-by-layer^1–14^. This approach offers a number of distinct advantages over conventional metal fabrication, including rapid prototyping, efficient material utilization, fabrication of complex geometries incompatible with machining or molding techniques, and applicability to materials which may exhibit machining difficulties, such as titanium^4,8–12,14^. However, unlike conventional manufacturing techniques, the LPBF process is characterized by rapid melting and resolidification of small volumes of material that results in large thermal gradients both temporally and spatially, and consequently cooling rates that can be orders of magnitude higher than in conventional casting^10,15,16^. Such large localized heating and cooling rates largely control the resulting microstructure, grain size and orientation, can lead to large residual stresses in some materials, and may also result in compositional segregation or formation of non-equilibrium trapped phases^4,17–20^. Therefore quantifying these cooling rates and correlating them to the characteristics of the final build is an important step towards informing theoretical and process models that can predict and potentially control such stresses and inhomogeneities that are generally undesirable, especially in critical components.

For this study, we focused on Ti-6Al-4V (Ti-64, 90%Ti, 6% Al, 4%V - by weight) as our material system. The high strength, light weight, and excellent corrosion resistance inherent to titanium and its alloys has led to their successful application to a wide range of aerospace, automotive, marine, power generation, and biomedical components. Ti-64, in particular, exhibits mechanical properties suitable for many critical applications requiring high strength to weight ratio as well as excellent biocompatibility^9,21,22^. Titanium commonly exists in two main allotropic forms, the room temperature hexagonal close-packed *α*-phase and the high temperature body-centered cubic *β*-phase^23–25^. Alloying with different elements can stabilize these different allotropes, enabling the design of microstructures with tailored mechanical properties. Ti-64 is such an *α*-*β* alloy, being composed of both phases at room temperature, and exhibiting an equilibrium phase diagram only slightly modified from pure titanium, with an *α* to *β* phase transition at 1270 K and a melting point of 1870 K^23,25^. As with other metallic components, the microstructure and phase in Ti-64 is intimately linked to the thermal histories during the manufacturing process and is well characterized for conventional manufacturing^26–29^. For LPBF fabrications on the other hand, where Ti-64 is commonly used, the link between build parameters, such as the laser speed and power, and the resulting phase and microstructure is tenuous and mostly derived from trial and error methods.

Significant computational effort is being directed at modeling the AM process, but experimental results describing the dynamic processes involved have derived mostly from optical approaches that are largely insensitive to subsurface effects^4,30–32^. Consequently, these methods cannot track the subsurface thermal evolution which is critical to understand the spatial evolution of microstructure in additively manufactured metals^33–36^. While optical pyrometry and thermal emission imaging can track temperatures, they are only sensitive to the surface, and thus, bulk temperatures must be inferred from models^19,37–45^. Similarly, thermocouples, as used in some studies, only measure temperatures relatively far from the hottest regions during this process and are inherently slow^4,46^. A number of recent studies have employed *in situ* X-ray probes to study the subsurface dynamics during AM processing, but they have either focused on imaging, or have been conducted in different spatial or temporal domains than that considered here^15,47,48^. Here, we demonstrate the use of *in situ* X-ray diffraction to monitor the subsurface structural evolution of the crystalline phases in Ti-64 as it cools below the solidification temperature to the *β*-transus following laser melting. We show how such data can be used to extract instantaneous bulk cooling rates of the resolidified material for the regime between resolidification and solid state transformation to the *α*-phase. We find that these rates increase monotonically from 2 × 10^4^ to 7 × 10^4^ K/s with decreasing laser power, all other scan conditions (such as laser scan speed, powder bed height, X-ray probe position) being held fixed. Furthermore, we find correlations between the cooling rates and and structural properties, specifically residual strain and grain size, of the *β*-titanium phase but no clear effect on the *α*-titanium phase, and point to possible mechanisms that give rise to the observed correlations.

## Results

**Figure 1:**
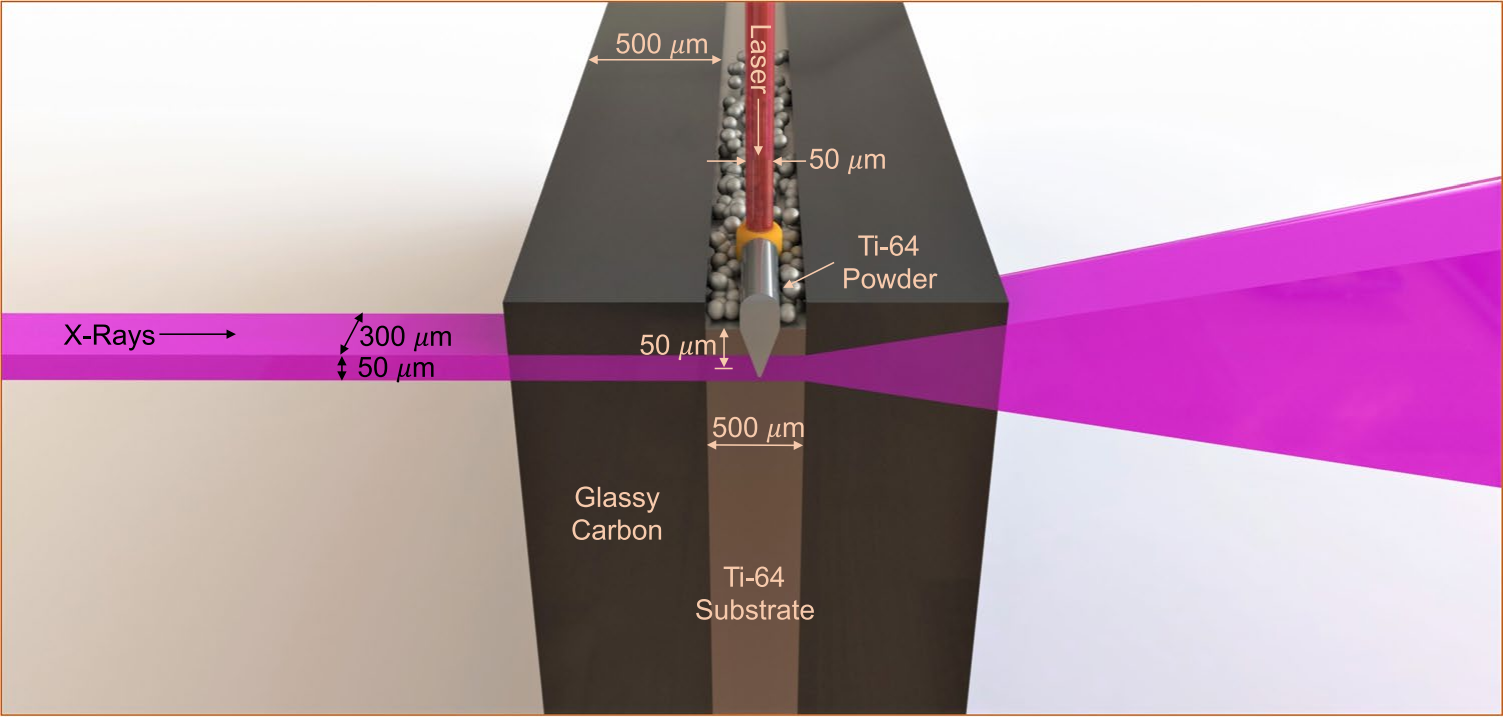
Schematic of experimental geometry showing the relevant dimensions. The dimensions are not to scale.

**Figure 2:**
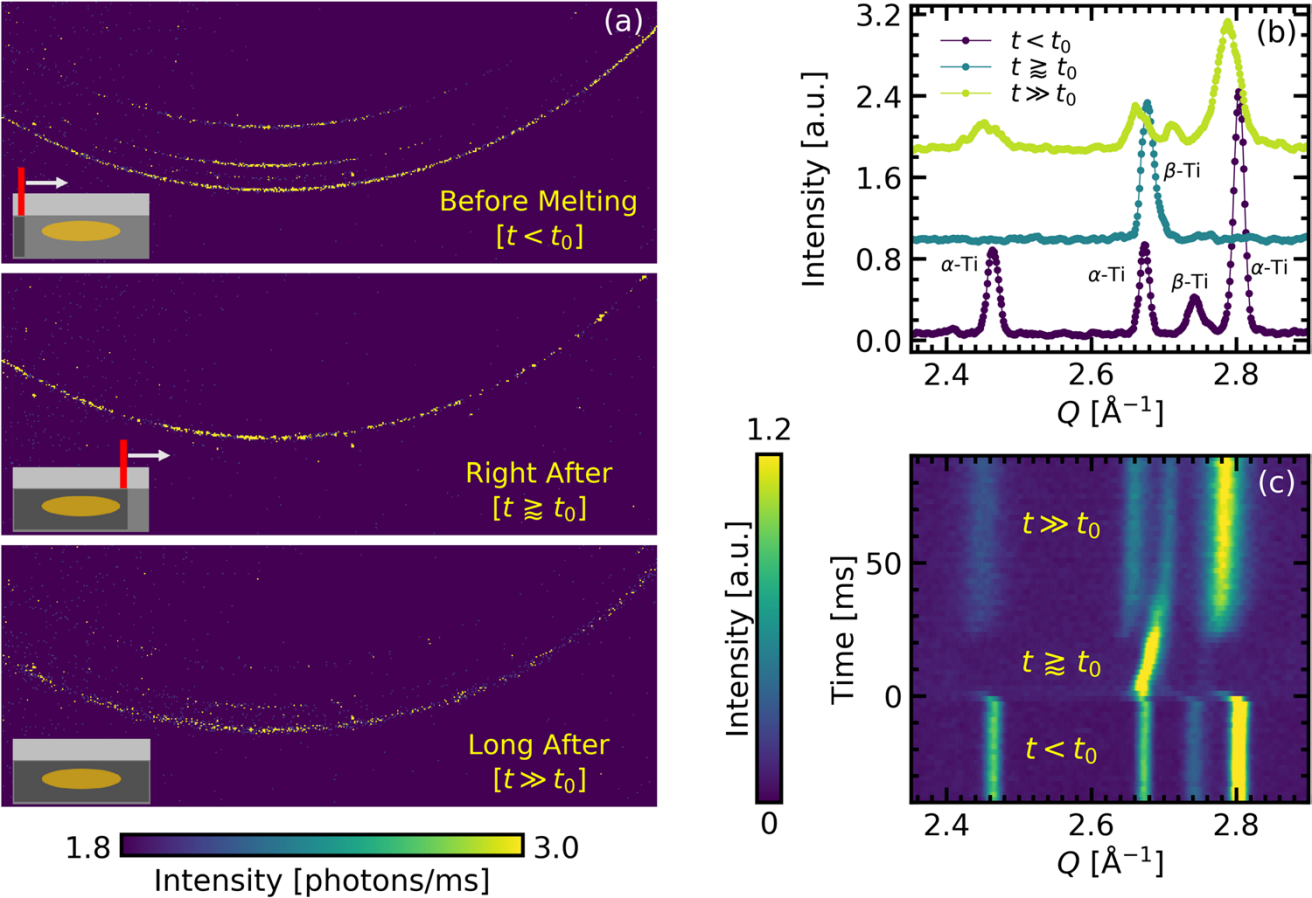
Diffraction data, collected at 500 Hz and 1 ms acquisition time per frame. Data is for build parameters of 200 W laser power, 144 mm/s scan speed, and X-ray spot size 50 *μ*m below the powder substrate interface. (**a**) 2D Diffraction patterns 10 ms before (top), 4 ms after (middle), and 80 ms after (bottom) the laser scan. The insets show the positions of the laser relative to the X-ray probed region schematically at these times. (**b**) Corresponding azimuthally integrated intensities as a function of *Q* in blue, green and yellow respectively. (**c**) Integrated intensities plotted as a function of time on the vertical axis to show the evolution of the diffraction peaks during the LPBF process. The intensities for (**a**,**c**) are encoded by color with the scales indicated by the respective color bars.

The experimental configuration is depicted in Fig. 1 and detailed in the methods section. Figure 2 shows typical diffraction data collected from a 500 *μ*m-thick Ti-64 substrate with a ∼50 *μ*m thick powder layer. These data were collected at a sampling rate of 500 Hz and 1 ms acquisition time per frame, with a 200 W laser scanned at 144 mm/s, and the X-ray beam center positioned ∼50 *μ*m below the powder substrate interface. This X-ray probe position simplifies analysis by precluding diffracted intensity contributions from flying spatter or powder particles. At the same time, since the melt depression and melt pool extend up to at least 100 *μ*m below the powder substrate interface^15,49^ [Also see Supplemental Fig. S3], the volume probed provides relevant information about the phase and microstructural evolution within a localized region undergoing melting and resolidification.

Figure 2a shows 2D diffractograms ∼10 ms before (top panel), ∼4  ms after (middle panel), and ∼80  ms after (bottom panel) the laser exposure. Azimuthally integrated intensities of these 2D diffraction patterns are plotted in Fig. 2b. Prior to laser melting, we see three clear *α*-Ti peaks ((100), (002), and (101)), and a small (110) *β*-Ti peak between the (002) and (101) *α*-Ti reflections, indicating that the unprocessed sample comprises a mix of *α* and *β* phases as expected for conventionally produced Ti-64 at room temperature^33–36^. About 4 ms after the laser passes the probed volume (Fig. 2a middle panel, and Fig. 2b green circles), we see that most of the diffracted intensity is concentrated in one peak that we identify as the (110) *β*-Ti peak, implying that most of the probed volume at this point is above the *β*-transus temperature. The position of the *β*-Ti peak right after the laser is significantly shifted towards smaller scattering vector, $Q=4\pi \,\sin \,\theta /\lambda$, which we attribute to thermal expansion of the crystal lattice. We also see a high *Q* tail to the *β*-Ti peak consistent with the variation in lattice parameters from the non-uniform temperature distribution within the probed volume that arises due to thermal gradients between the melted zone (MZ), the nearby unmelted but heat affected zone (HAZ), and the farther away unaffected zone. The kymograph in Fig. 2c shows the azimuthally integrated intensities encoded by color and stacked along the vertical time axis to follow the evolution of the diffracted peaks during the LPBF process. We see that for about 30 ms following laser melting, the diffraction intensity is mainly concentrated in the *β*-Ti peak. Over this time, the position of the *β*-Ti peak that had abruptly shifted to a smaller *Q* value immediately following laser melting, gradually shifts to higher *Q* as the lattice contracts upon cooling. After about 30 ms, a substantial fraction of the probed volume has cooled below the *β*-transus, as evidenced by the abrupt reappearance of the *α*-Ti peaks that grow in intensity as that of the *β*-Ti peak diminishes. After ∼100 ms, the peak positions and intensities remain nearly constant indicating the sample has reached thermomechanical equilibrium and the diffracted intensity is now redistributed between the low temperature *α* and *β* phases. We note here that previous work has shown that LPBF of Ti-64 typically leads to a martensitic (*α*′) phase due to the rapid cooling^9,16,29,50,51^. However, we cannot differentiate between the *α* and *α*′ phases from the diffraction signals, and for brevity we will henceforth refer to this phase as *α*-Ti.

**Figure 3:**
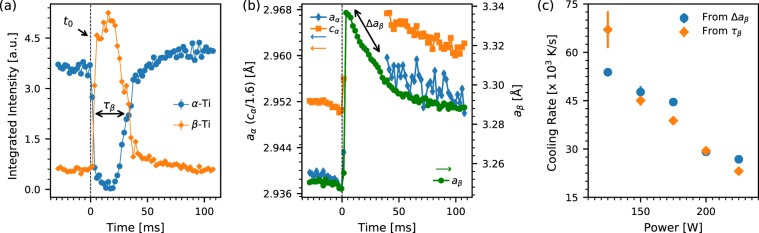
*In situ* cooling rates, calculated for data collected at 500 Hz frame rate and 1 ms acquisition time per frame. (**a**) Time evolution of scale factors of the *α*-Ti (blue circles) and *β*-Ti (orange diamonds) crystalline phases for laser power of 225 W and speed 144 mm/s. The drop in the *α*-Ti peak intensity coincides with the laser indicated by the dashed black line. Also, shown in the figure is the time period (*τ*_*β*_) for which the phase fraction is mainly *β*-Ti. The solid lines are guides for the eye. (**b**) Lattice parameters of the *α*-Ti *a*- and *c*-parameters (blue diamonds and orange squares respectively) and *β*-Ti phase (green circles) as a function of time for same process parameters as (**a**). The *c*-lattice parameter of the *α*-Ti phase is divided by the approximate *c*/*a* ratio of 1.6 for the pristine material. The change in the lattice parameters of the *β*-Ti phase over *τ*_*β*_ is indicated as Δ*a*_*β*_. The lattice parameters for the *α*-Ti phase are not shown during *τ*_*β*_ because of the large uncertainties in this time range. (**c**) Cooling rates for a range of laser powers calculated from rate of lattice relaxation (blue circles), and *β*-Ti lifetime, *τ*_*β*_, (orange diamonds).

We have performed fitting analysis in order to quantitatively track the evolution of the crystalline titanium phases during the LPBF process. Diffraction patterns were modelled as a combination of *α*- and *β*-Ti phases. The full time series was fit sequentially in three time segments: prior to the laser the data were fit to a single *α*- and single *β*-phase, subsequent to the laser melting and prior to traversing the *β*-transus the data were fit to a single *α*-phase representing the HAZ and two distinct *β*-phases represnting the MZ and HAZ respectively, and after the *β*-transus as two distinct *α*- and *β* phases for the MZ and HAZ. Phases were modelled with fixed atomic coordinates and thermal parameters but with variable scale factors and lattice parameters. Results of these refinements, shown in Fig. 3a,b, were used to extract cooling rates for the temperature range between resolidification but above the solid state transformation from *β*- to *α*-phases.

The first method involves determining the *β*-lifetime (*τ*_*β*_) as shown in Fig. 3a, that we define as the time immediately following the laser induced melting and resolidification for which the *β*-Ti phase is the predominant phase. As we see in Fig. 3a, the value of *τ*_*β*_ can be determined by following the time evolution of the intensities of the *β*-Ti peaks, or equivalently the *α*-Ti peaks. An asymmetric rectangular function with error functions to define the up and down steps is fit to the peak intensity, and *τ*_*β*_ is calculated as the distance between the centers of the two error functions. This corresponds to the time required for the probed sample volume to cool down from the melting point (∼1870 K) to the *β*-transus temperature (∼1270 K), below which the diffracted signal is again dominated by the primary *α*-Ti phase, and thus we obtain the average cooling rate over this temperature range. This method makes two simplifying assumptions. First, it does not take into account undercooling that might lower either the solidification or solid state transition temperatures. As reported in another study^47^, undercooling lowers the melting temperature by ∼40 K, and the error introduced by a change of this magnitude is accounted for by the error bars. Second, it does not explicitly take into account the instantaneous thermal gradients within the probed volume. Here, we make the assumption that even though the entire probed volume does not transition simultaneously across the *β*-transus, the half maximum points of the error step functions used here to fit the intensity profiles during the transitions are a good approximation to the volumetric average.

The second method estimates the cooling rate from the rate of change of the *β*-Ti lattice constants as the sample cools through the *β*-Ti only phase. The negative shift in peak positions (Δa_*β*_) during *τ*_*β*_, as shown in Fig. 3b, is due to lattice contraction as the sample cools. If we attribute this contraction primarily to thermal contraction on cooling, we can use the known coefficient of thermal expansion for the relevant temperature range and fit the following expression obtained by applying Newton’s law of cooling:

1 $${a}_{\beta }(t)={a}_{\beta }({T}_{0})\times [1-C{T}_{0}(1-{e}^{-Rt})]$$

to the value of *a*_*β*_ during *τ*_*β*_ to obtain an estimate of the cooling rate, R. Here, ${a}_{\beta }({T}_{0})$ is the *β*-phase lattice parameter at temperature *T*_0_ and time *t* = 0, *a*_*β*_(*t*) is the *β*-phase lattice parameter at a later time *t*, *C* is the relevant coefficient of thermal expansion ($\sim 9\times {10}^{-6}$/K)^52^ and *R* is the rate constant for cooling. By setting the melting temperature (1870 K) as *T*_0_, we get the cooling rate at the melting point and approximate it as the average cooling rate through the *β*-transus (the cooling rate is fairly constant over this range). This approach discounts contributions from residual strain and assumes that the dominant contribution to the instantaneous change in lattice parameter is from thermal expansion given the high cooling rate. The potential effect of compositional variations occurring within the probed volume during this period, *τ*_*β*_, is also disregarded since diffusive transformations are generally suppressed at such timescales^9,16,29,50,51^, and we assume no overall loss of material during this process.

The average cooling rates over the temperature range between the resolidification and the *β* to *α* transformation temperature are shown in Fig. 3c as a function of laser power with a constant laser scan speed of 144 mm/s and all other printing parameters held fixed, extracted using the two methods described above. The blue circles are cooling rates based on changes in *β*-Ti lattice constant, while the orange diamonds show the values obtained from *τ*_*β*_ using the intensity time dependence of the *α*-Ti phase fraction. These cooling rates obtained by different methods are consistent within statistical uncertainties, which indicates these are robust estimates of the cooling rates despite the caveats listed above for each of the methods. There is a clear inverse relationship between laser power and cooling rate, where the highest cooling rate of $\sim 6.5\times {10}^{4}$ K/s corresponds to the lowest laser power (125 W), decreasing nearly linearly to $\sim 2.5\times {10}^{4}$ K/s as the power is ramped up to 225 W.

**Figure 4:**
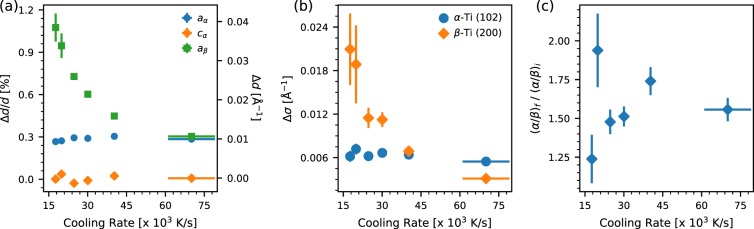
Correlating the cooling rates to changes in structural parameters and phase fraction of final build measured after the laser exposure. The change in (**a**) lattice parameters (both relative and absolute), (**b**) peak widths, and (**c**) phase fraction (*α*-Ti/*β*-Ti) are shown as a function of cooling rate calculated by taking the average of the cooling rates from the two methods outlined in the text.

Comparing the final state the the initial state (as in Fig. 2b), we observe a number of changes to the material at ambient conditions after LPBF. Even after a significant relaxation time (>1 s) allowing the material to return to thermomechanical equilibrium, the diffraction peaks show a systematic shift towards lower *Q*, indicating a change in the bulk lattice constant. We also observe that the peaks become broadened and, evident from fitting the data, the relative peak intensities of the *α*- and *β*-phases is changed. The relative changes of lattice parameter, peak widths, and phase fraction of the printed track to the pristine material are shown in Fig. 4 as a function of the derived cooling rates. There is a significant shift in the lattice parameter of the *β*-phase to larger values subsequent to printing of the track which increases with slower cooling rate (Fig. 4a). The lattice parameter of the *α*-phase is independent of cooling rate for the conditions studied here and shows a small expansion in the *a*, *b*-plane and none along the *c*-axis. The width of the diffraction peaks also broadens after laser printing, with a small but noticable change for the *α*-phase peaks which is again independent of cooling rate while the *β*-phase diffraction peaks exhibit increased broadening at lower cooling rates (Fig. 4b). Following the LPBF process, the material is enriched in *α*-phase fraction by an amount which is uncorrelated with cooling rates above the *β*-transus (Fig. 4c).

## Discussion

The cooling rates obtained over temperatures between resolidification and the *β*-transus by different analytical methods are consistent within statistical uncertainties. This suggests that any undercooling of the melting transition and the *β*- to *α*-phase transition are small or at least comparable, and that any strain which develops over this cooling regime has a negligible effect on lattice parameters relative to the thermal expansion. As significant lattice shifts remain present in the final diffraction patterns, this also suggests that lattice strains only become significant after the material has cooled to below the *β*-transus temperature, outside of the regime for which we have extracted cooling rates. We attribute the trend in cooling rate to an increase in the heated volume, resulting in smaller thermal gradients and less effectiveness of the surrounding material as a heat sink. Cross sectional SEM images (Supplemental Fig. S4) show a largely similar width of the melt pool at the depth measured by the X-ray beam. Measurements of the surface track widths, which are a reasonable proxy for the width of the melt pool 50 *μ*m below the interface^53^, are also largely invariant for the range of laser powers considered. The melted and resolidified region accounts for roughly 25% of the volume probed by the X-ray beam for all laser powers, suggesting that changes to the resolidified volume alone are not sufficient to account for the observed trend in cooling rate. The heat affected zone, in contrast, grows from roughly 30% to 70% of the probed volume as a function of increasing laser power. The decreased cooling rate with increasing laser power causes this enlargement of the HAZ. This leads to a higher temperature of the heat sink as the combined size of the melt pool and the heat affected zone grow with larger amounts of deposited energy, slowing the rate at which thermal energy can be extracted from the resolidified region. The depth of the melt pool will also affect the thermal transport along the vertical direction, slowing heat extraction as the melt pool becomes deeper for the same reason - a higher average temperature heat sink. Finally, we cannot rule out effects from non-ideal thermal boundary conditions in our system at higher laser powers. Even though the thermal conductivities are comparable for Ti-64 and glassy carbon^24,54^, the interface between the two materials introduces additional thermal resistance that likely has an impact on the heat sink effectiveness.

To correlate the cooling rate with the final build, we examined its effect on several characteristics of the final microstructure as summarized in Fig. 4. These plots show the relative changes between the initial pre-laser state and the final state measured ∼1 s after laser exposure when we can safely assume thermal equilibrium. Figure 4a plots the change in the lattice parameters of the *α*-Ti and *β*-Ti phases as a function of cooling rate. We observe an overall expansion of the lattice for both phases, although only the *β*-phase shows a marked cooling rate dependence. The change in the *β*-Ti phase has its highest value of ∼1% at the lowest cooling rate and decreases monotonically with increasing cooling rate to a minimum value of ∼0.3%. The *α*-Ti phase on the other hand shows a roughly constant 0.3% expansion along the *a*- and *b*-axes with no dependence on the cooling rate and no change in the *c*-axis. As noted earlier, this expansion may be attributed to residual tensile strain and/or compositional change. It is not straightforward to separate the relative contributions of these two effects from diffraction data alone.

We performed energy dispersive spectroscopy (EDS) measurements on the cross-sectioned samples to examine the compositional variation in the different regions of the probed volume. Supplemental Figs. S2 and S3, and the associated tables, show that while the *β*-phase in the HAZ and the region far from the HAZ are significantly V-rich (∼12.5% by wt.), the MZ has a far more uniform V distribution (∼3.6%) as the vanadium is redistributed over the entire melt volume and does not have time to partition to the *β*-phase due to the high cooling rate. Since the lattice parameter of *β*-phase increases with decreasing V content^23,52^, at least part of the *β* lattice expansion can be ascribed to this compositional change. However, if we again consider the melt pool widths, the unchanged MZ fraction cannot account for the trend in lattice change alone, implying that the observed trend is due to increasing residual strain in the *β*-phase with decreasing cooling rates. The time dependent diffraction data shows that there is a period where the probed volume is almost all in the *β*-Ti phase, above the *β*-transus. As the material cools below the *β*-transus, a large fraction of this material will undergo a transformation to the *α*-Ti phase, leaving small regions of *β*-Ti in an *α*-Ti matrix. The volumetric contraction of the *β*-Ti phase upon cooling is significantly greater than that of the *α*-Ti phase, which will generate a tensile strain in the untransformed islands of *β*-Ti^48^. The details of this *β* to *α* transformation determine the level of this tensile strain which develops. Although studied for cooling rates orders of magnitude slower than observed here, there is evidence that the degree of transformation at a given temperature will be more advanced for a slower cooling rate^16,27^. This would suggest that slower cooling rates produce a more complete *α*-Ti matrix at a higher temperature, leading to a greater temperature change for the mixed phase material and a greater thermal expansion mismatch producing a larger strain in the residual *β*-Ti material. Therefore, while some of the observed lattice shift for the *β*-Ti phase may be due to a lower vanadium content, the cooling rate dependence is likely a result of different levels of strain which build during the cooling process following transformation to the *α*-Ti phase (note that this would not affect the cooling rate determination, which deals with temperatures above this phase transition). The lattice shift observed for the *α*-Ti phase may be due to a compositional change, although the slight enrichment in vanadium, which is expected, would generate a lattice contraction, not the observed expansion, or to residual strains which develop as a result of the martensitic transformation from the *β*-phase.

The diffraction peaks show a systematic broadening following LPBF compared to the starting material, as shown in Fig. 4b. While the widths of both *α*-Ti and *β*-Ti peaks increase after resolidification, the *β*-Ti peak widths also increase systematically with decreasing cooling rate. Diffraction peak widths, ignoring instrumental contributions, are affected by crystallite grain size and mosaicity, inhomogeneous microstrain produced by defects, anisotropic or inhomogeneous macrostrain, and chemical inhomogeneity. As a result of the rapid quenching of the material, especially through the *β*-transus, it is reasonable to expect a finely grained microstructure with significant levels of defects and residual strain. The EDS measurements suggest that in the resolidified region the elemental composition is homogeneous. However, in the HAZ and the surrounding material, there still exists a large degree of phase partitioning, especially of the vanadium. The difference in composition, and thus lattice paramters, over the volume sampled by the X-ray beam is likely to lead to some degree of peak broadening, as would the small grain size and microstrain induced by any defects. The cooling rate dependence on the *β*-Ti phase peak widths is unlikely to be due to these effects, however, as the size of the compositionally homogeneous resolidified region is invariant with cooling rate and the quenching is too rapid to allow for annealing of defects or signficant grain growth. This cooling rate dependence, instead, is likely due to the same mechanism which gives rise to the cooling rate dependent strain observed in the lattice parameters. Inhomogeneity in strain, when volume averaged, will show broader diffraction peaks. It is also possible that the larger strains developed at slower cooling rates also have a larger variation, leading to the broader observed diffraction peaks.

The ratio of the relative fractions of *α*-Ti and *β*-Ti phases before and after LPBF are shown in Fig. 4c. We observed a consistent decrease in the *β*-phase fraction of the final material at all laser scan parameters. This observation is consistent with the more homogenous, and effectively reduced, redistribution of the *β*-stabilizing V in the resolidified material, which is itself invariant in fraction of the probed volume. No systematic dependence of this change on the cooling rate is observed, implying that while the microstructural properties of the *α*-Ti and *β*-Ti phases are intimately related to the cooling rate, the phase fraction itself remains unaffected over the cooling rates observed here. Literature reports of the *α* to *β* ratio in LPBF parts have reported purely *α*-phase^6,17,18^ while others report an *α* + *β* microstructure^20,55^. Our results support a mixed *α* + *β* microstructure, however we cannot rule out the possibility that this is dependent on laser scan speed and power where the range investigated here confines the result to a single laser pass regime. Furthermore, since this work focuses on single layer melting, we do not directly investigate if this variation in observed microstructure arises from initial solidification conditions, the subsequent cyclic rapid heating and cooling enforced by the LPBF process during a full build, or some interaction between these process parameters. We also cannot rule out texture effects in the small amount of *β*-phase present in the final volume redistributing the scattering intensity into unmeasured directions.

## Conclusions

This work employs high speed *in situ* diffraction to track the evolution of microstructural and phase characteristics of Ti-64 alloy during the LPBF process and from that extract bulk cooling rates of the material following resolidification of the melt. We show that the cooling rate depends systematically on LPBF process parameters such as laser power and correlate these cooling rates to the microstructural features of the final material. We find that the resultant *β*-phase component bears the majority of the residual strain in the material, which is reduced for faster cooling rates. We also observe a uniform redistribution of V in the resolidified region in contrast to the starting base material which correlates to a reduction in *β*-phase fraction. These insights are crucial to formulate a sound theoretical underpinning of the LPBF process in general, and to understand how the properties of AM manufactured Ti-64 components can be predicted.

## Methods

The LPBF process was carried out using a system equipped with an IPG Photonics fiber laser that produces a 1070 nm laser beam with power that can be varied between 20–500 W. This is coupled with a 3-axis galvanometer scanning mirror system that focuses and scans the process laser across the powder/substrate. The intensity distribution of the laser is Gaussian and is focused to a nominal spot size of 50 *μ*m D4 *σ*. The entire setup is enclosed in a vacuum chamber with beryllium windows to allow transmission of X-rays and is filled with argon to prevent oxidation of the powder during the LPBF process. Further details about the system can be found in Calta *et al*.^49^.

The samples consisted of Ti-64 substrates machined from sheet (TMS Titanium, Grade 5) with Ti-64 powder with particle diameter of 30 ± 10 *μ*m (Additive Metal Alloys) on top, sandwiched between glassy carbon windows. We used a substrate thickness of 500 *μ*m along the X-ray beam direction, and the powder bed depth was 50–70 *μ*m. The thickness of the sample along the beam direction is chosen as a compromise between an idealized system without artificially imposed thermal boundary constraints and one that would be thin enough to allow sufficient X-ray transmission.

The X-ray diffraction experiments were carried out at beamline 10-2, Stanford Synchrotron Radiation Lightsource, SLAC National Accelerator Laboratory using 20 keV photons (0.6199 Å wavelength). The size of the X-ray beam that defines the spatial resolution of the probed volume was defined by slits before the sample. The data shown here were collected using 50 *μ*m (vertical) × 300 *μ*m (horizontal) slits. Part of the data shown here were collected using a Pilatus 100 K detector (Dectris Ltd., Switzerland) at 500 Hz, and the rest with an Eiger 1 M area detector (Dectris Ltd., Switzerland) at 1 kHz (see Supplemental Movie for an example of the time resolved area diffraction data). The detector positions were set to provide the maximum possible azimuthal coverage of at least three *α*-Ti ((100), (002), and (101) reflections from hexagonal close packed (hcp, space group $P{6}_{3}/mmc$)) and one *β*-Ti ((110) reflection from body centered cubic (bcc, space group $Im\bar{3}m$)) diffraction rings, while also achieving sufficient *Q* resolution to distinguish these peaks (${Q}_{max}=2.98$ Å^−1^ with a *Q* resolution $\Delta Q/Q=0.005$ Å^−1^).

## Supplementary information

Diffraction Movie.

Supplemental Information.
