# Supplementary material for: Subsurface Cooling Rates and Microstructural Response during Laser Based Metal Additive Manufacturing
Source: Sci Rep. 2020 Feb 6;10:1981. doi: 10.1038/s41598-020-58598-z (PMC7005153; doi:10.1038/s41598-020-58598-z)
Supplement: Supplementary file 2 — Supplemental Information. [file 41598_2020_58598_MOESM2_ESM.pdf]

# Subsurface Cooling Rates and Microstructural Response during Laser Based Metal Additive Manufacturing

**Vivek Thampy[1,*], Anthony Y. Fong[1], Nicholas P. Calta[2], Jenny Wang[2], Aiden A. Martin[2], Philip J. Depond[2], Andrew M. Kiss[1], Gabe Guss[3], Qingfeng Xing[4], Ryan T. Ott[4], Anthony van Buuren[2], Michael F. Toney[1], Johanna Nelson Weker[1], Matthew J. Kramer[4], Manyalibo J. Matthews[2], Christopher J. Tassone[1], and Kevin H. Stone[1,*]**

[1]Stanford Synchrotron Radiation Lightsource, SLAC National Accelerator Laboratory, CA 94025, USA
[2]Physical and Life Sciences Directorate, Lawrence Livermore National Laboratory, CA 94550, USA
[3]Engineering Directorate, Lawrence Livermore National Laboratory, CA 94550, USA
[4]Division of Materials Science and Engineering, Ames National Laboratory, IA 50011, USA
[*]vthampy@slac.stanford.edu, khstone@slac.stanford.edu

## Supplemental Information

In addition to the supplemental information provided here, we have included a movie of the time resolved X-ray diffraction. The movie is comprised of area diffraction data collected using an Eiger 1M detector at a 1kHz frame rate. The data are collected $50\mu$m below the substrate/powder interface with a laser power of 225W and a scan speed of 144mm/s.

Here we include *ex situ* optical microscopy data that shows the surface track widths as a function of power, scanning electron microscopy (SEM) images of the cross sections, and energy dispersive spectroscopy (EDS) analysis performed to obtain the variations in the elemental compositions variations in the melted zone (MZ), heat affected zone (HAZ) and the unaffected zone (UAZ) far from the HAZ.

| Spectrum → | Spec 3 | Spec 4 | Spec 5 | Spec 6 | Spec 7 |
|---|---|---|---|---|---|
| Al | 3.10 | 3.53 | 5.95 | 6.07 | 6.00 |
| V | 14.26 | 12.52 | 2.55 | 2.32 | 2.51 |
| Total (wt.%) | 100.00 | 100.00 | 100.00 | 100.00 | 100.00 |

**Table 1.** Elemental composition of various regions in the BSE map of the base substrate obtained by energy dispersive spectroscopy (EDS) analysis of selected areas from Fig. 2. Spectrum 3 and 4 are from the vanadium rich $\beta$-Ti while spectrum 5 - 7 are from the vanadium poor $\alpha$-Ti regions. The Al was standardized using $Al_2O_3$ while the Ti and V were standardized using elemental standards. The data was acquired using an Oxford AztecHKL system on a FEI Teneo Field emission SEM operating at 15 keV using standard package for ZAF corrections. Values are in wt.% with uncertainty of $\sim 5\%$.

| Spectrum → | Spec 8 | Spec 9 | Spec 10 | Spec 11 | Spec 12 | Spec 13 | Spec 14 | Spec 15 |
|---|---|---|---|---|---|---|---|---|
| Al | 5.97 | 5.95 | 5.98 | 5.98 | 5.74 | 5.74 | 5.75 | 5.73 |
| V | 2.48 | 2.70 | 2.41 | 2.52 | 3.59 | 3.64 | 3.58 | 3.65 |
| Total (wt.%) | 100.00 | 100.00 | 100.00 | 100.00 | 100.00 | 100.00 | 100.00 | 100.00 |

**Table 2.** Elemental composition of various regions in the BSE map of printed track obtained by EDS analysis of selected areas from Fig. 3 showing that the composition of the $\alpha$-Ti composition (gray phase) is virtually unchanged between the base metal (spectrum 8) and the HAZ (spectra 9 - 11). However, the melted zone (spectra 12 - 15) show substantial increase in V. Data acquisition and analysis as described above. Further sampling of the composition of the melt pool from the surface to the bottom show virtually no change in the $\alpha$-Ti.

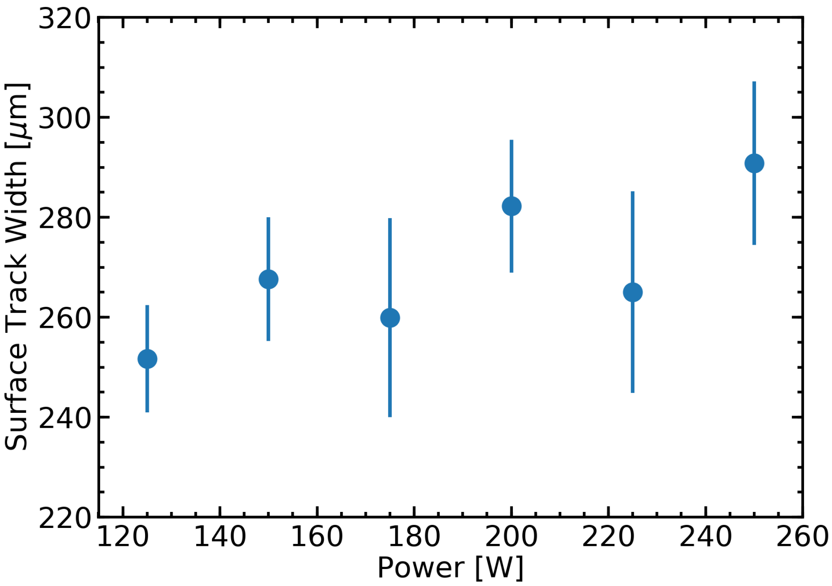


**Figure 1.** Surface track widths as a function of laser power. Track widths measured at the surface are measured for different laser powers with 144 mm/s scan speed measured *ex situ* using an optical microscope. These values and the error bars were obtained by taking the average and standard deviation of the track widths measured at three points close to the center of the track (the position of the X-ray probe.)

| Cooling Rate (x $10^3 K/s$) | Lattice Parameters: $a$ ($\alpha$-Ti) Before | $a$ ($\alpha$-Ti) After | $c$ ($\alpha$-Ti) Before | $c$ ($\alpha$-Ti) After | $a$ ($\beta$-Ti) Before | $a$ ($\beta$-Ti) After |
|---|---|---|---|---|---|---|
| 70(9) | 2.92 | 2.93 | 4.66 | 4.67 | 3.21 | 3.22 |
| 40(2) | 2.92 | 2.93 | 4.67 | 4.67 | 3.21 | 3.22 |
| 30(2) | 2.92 | 2.93 | 4.67 | 4.67 | 3.21 | 3.23 |
| 25(2) | 2.92 | 2.93 | 4.67 | 4.67 | 3.21 | 3.23 |
| 20(2) | 2.92 | 2.93 | 4.67 | 4.67 | 3.21 | 3.24 |
| 18(1) | 2.92 | 2.93 | 4.67 | 4.67 | 3.21 | 3.25 |

**Table 3.** Lattice parameters of the $\alpha$- and $\beta$-phases measured at room temperature before and after LPBF for the different cooling rates determined in this work.

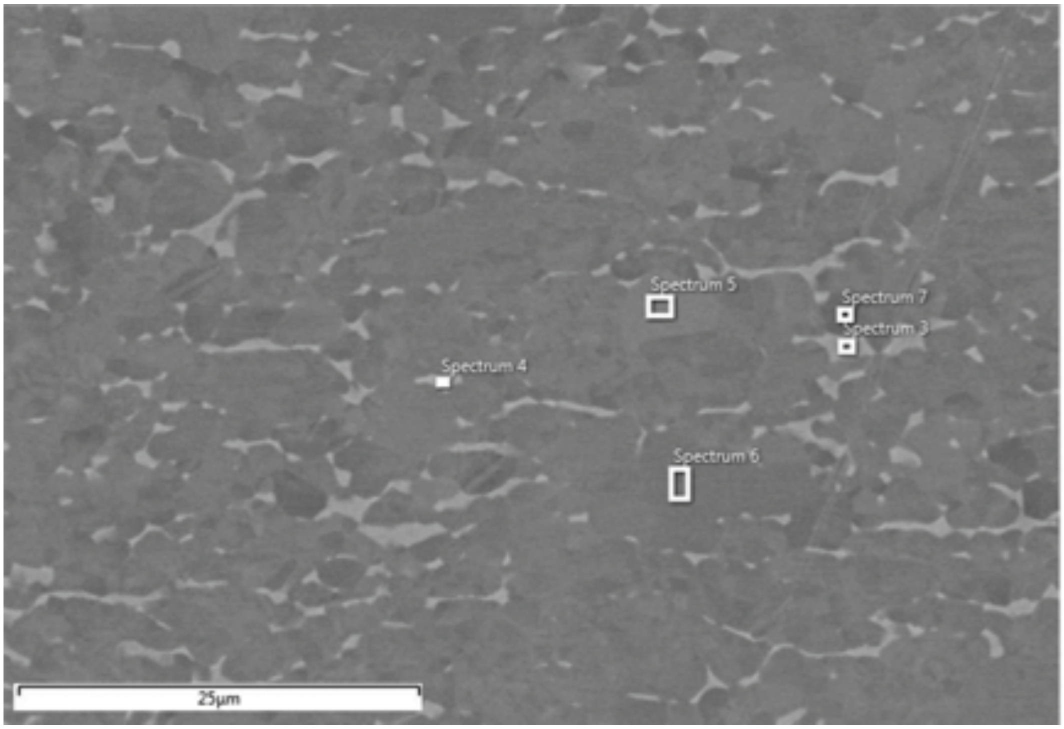


**Figure 2.** BSE image of the base substrate with elemental composition of various regions measured far from the heat affected zone showing the vanadium rich $\beta$-Ti (bright regions) and darker gray vanadium poor $\alpha$-Ti (see Table 1). The image was acquired using a FEI Teneo Field emission SEM operating at 15 keV.

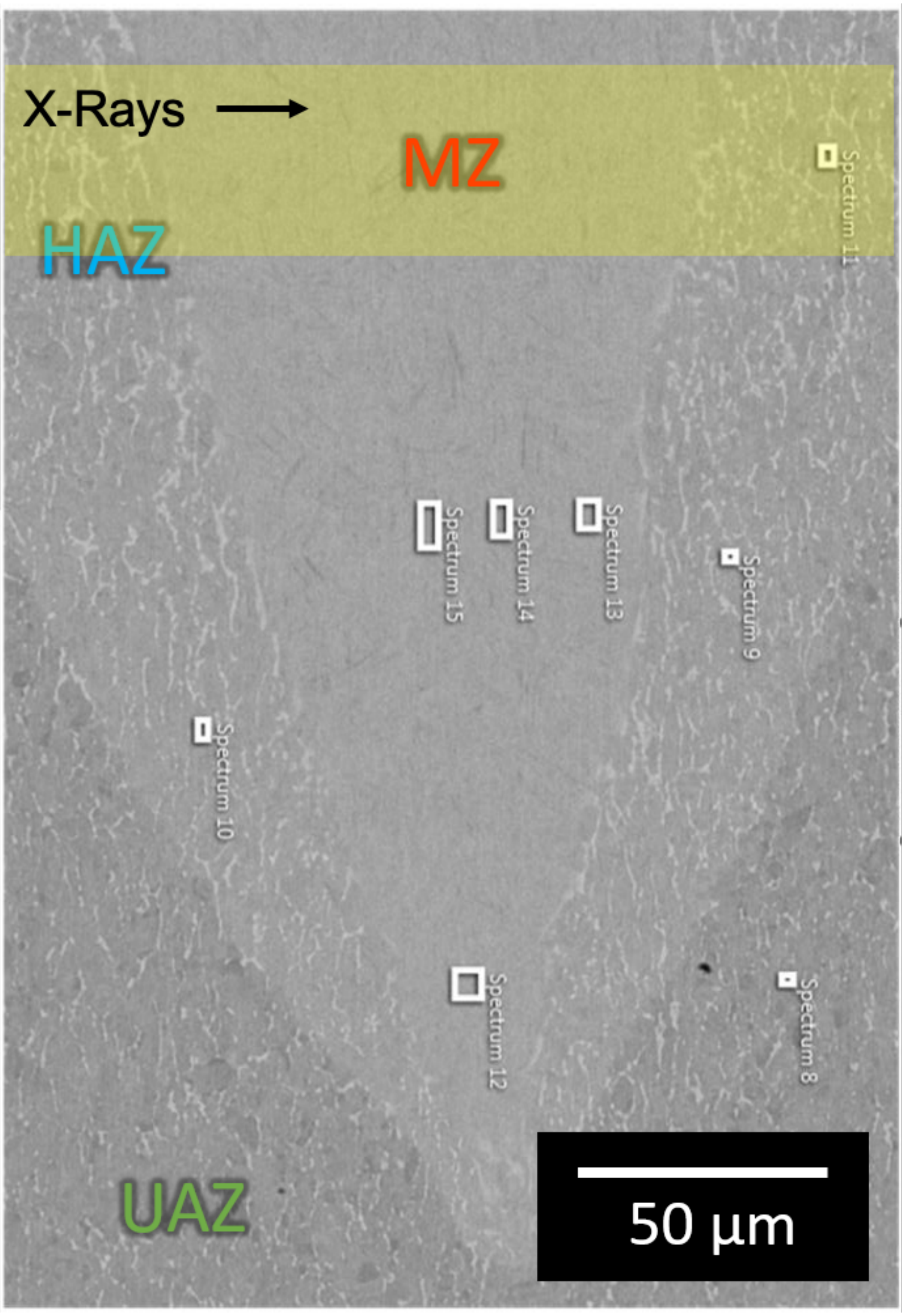


**Figure 3.** EDS analysis of the melted zone (MZ), heat affected zone (HAZ) and base substrate (UAZ) for a printed track with laser power of 225 W and 144 mm/s scan speed. The region probed by the X-Rays is highlighted in yellow. The slight variation in the gray scale between the base metal and the HAZ is most likely due to subtle changes in the microstructure since the EDS did not show substantial changes in the $\alpha$-Ti phase. The melt pool shows substantial changes in the microstructure and incorporation of more V into $\alpha$-Ti (Table S2.)

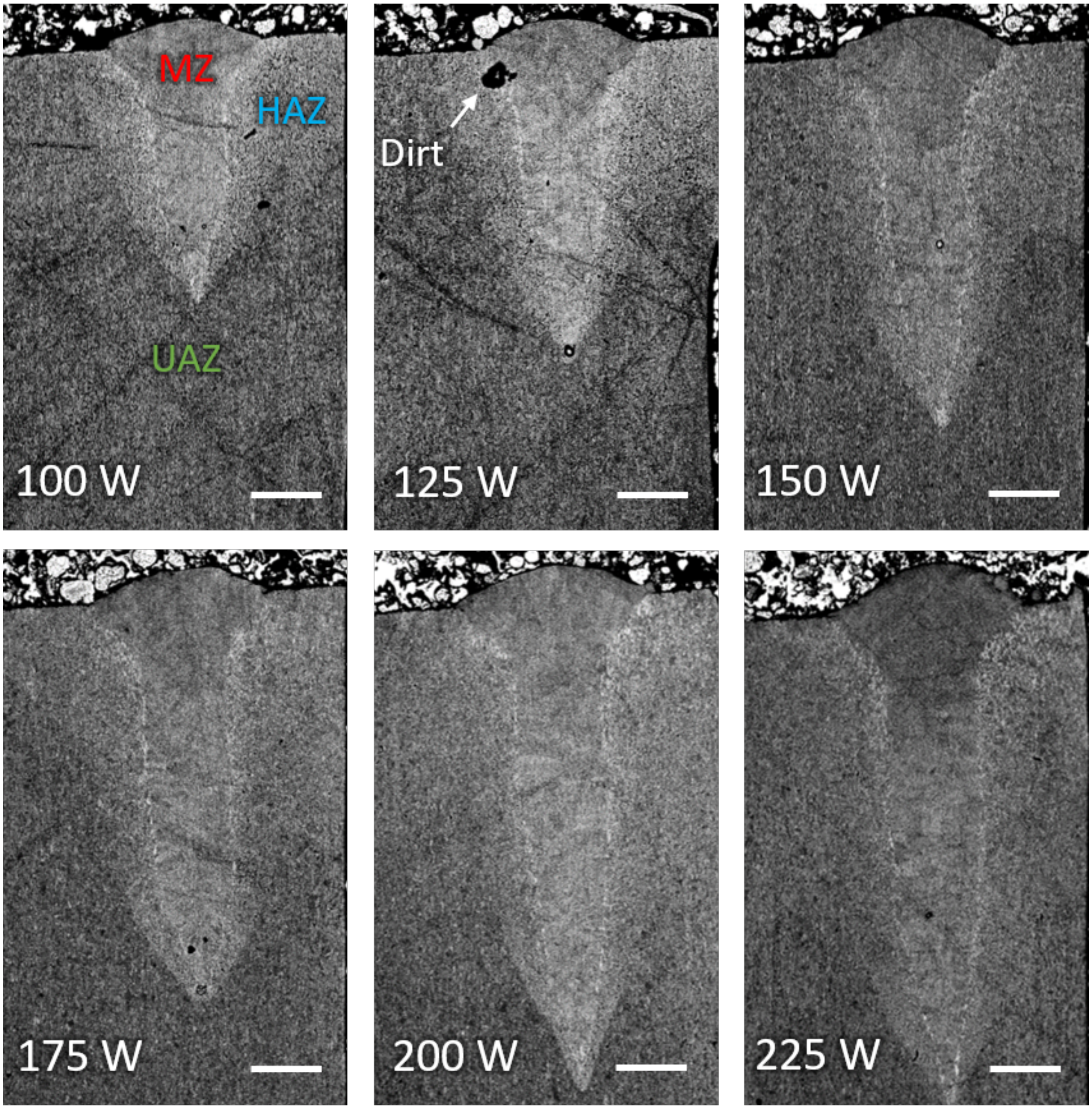


**Figure 4.** SEM backscattered electron (BSE) images of the printed tracks showing the MZ, HAZ and UAZ for different laser powers at 144 mm/s scan speed. Scale bar is 50 $\mu$m for all images.
